# Supplementary material for: Household costs, catastrophic out-of-pocket payments and impoverishment related to accessing surgical care in rural Ethiopia
Source: PLoS One. 2026 Feb 6;21(2):e0294215. doi: 10.1371/journal.pone.0294215 (PMC12880665; doi:10.1371/journal.pone.0294215)
Supplement: S1 Table — (DOCX) [file pone.0294215.s001.docx]

**Supplementary table 1: Household consumption expenditure cost categories (in Birr)**

| Expenditure categories | Mean (SD) | Percentage share |
| --- | --- | --- |
| Food | 5970.3(4400.3) | 43.8 |
| Household utilities | 2423.3(7981.7) | 17.7 |
| Big purchase | 1694.9(6830.9) | 12.4 |
| Healthcare(direct medical and non-medical) | 2876.1 (3848.9) | 21.2 |
| Total | 12964.7 (15286.1) |  |

*1 USD = 29.07 Birr (2019, purchasing power parity, 1 international dollars=10.74 Birr)*

*Food items (staple foods, vegetable, fruit, spices etc.), regular household expenses (electricity, water, cooking, renting, clothing, transport, etc.), Big purchase include (education, durable goods, cultural ceremonies, entertainment, tax.); health expenditure include expense on (outpatient consultation, medication, investigations, hospitalization, medical appliances, ambulance, hospital food, accommodation and transport cost related to surgical care)*
