# Supplementary material for: Household costs, catastrophic out-of-pocket payments and impoverishment related to accessing surgical care in rural Ethiopia
Source: PLoS One. 2026 Feb 6;21(2):e0294215. doi: 10.1371/journal.pone.0294215 (PMC12880665; doi:10.1371/journal.pone.0294215)
Supplement: S2 Table — (DOCX) [file pone.0294215.s002.docx]

**Supplementary Table 2: Poverty impact of Out-of-Pocket payments for surgical care**

| Poverty impact of OOP payments | Half(1/2) of median total consumption | Two -thirds(2/3) of median total consumption |
| --- | --- | --- |
| Poverty head count |  |  |
| Pre-payment head count ^X^ | 18.7% | 29.1% |
| Post payment head count ^Y^ | 29.1% | 48.3% |
| Absolute percentage point change(head count impact) ^Z^_(=Y-X)_ | 10.4% | 19.2% |
| Relative percentage change (=Z/X*100) | 55.6% | 65.9% |
| Poverty gaps |  |  |
| Prepayment poverty gap(Birr) ^X^ | 315.0 | 666.5 |
| Post payment poverty gap(Birr) ^Y^ | 604.9 | 1216.9 |
| Absolute point change(Birr))^Z^_(=Y-X)_ | 289.9 | 550.4 |
| Relative percentage change (=Z/X*100) | 92.0% | 82.5% |
| Normalized poverty gaps |  |  |
| Pre-payment normalized gap ^X^ | 6.6% | 10.4% |
| Mean positive pre-payment poverty gap(Birr) | 1686.4 | 2511.5 |
| Post-payment normalized gap ^Y^ | 12.6% | 19.1 |
| Mean positive post-payment poverty gap(Birr) | 2077.4 | 2516.8 |
| Absolute percentage point change (impact) ^Z^_(=Y-X)_ | 6.0 | 8.6 |
| Relative percentage change (=Z/X*100) | 90.9 | 82.7 |

*1 USD = 29.07 Birr (2019, purchasing power parity, 1 international dollars=10.74 Birr)*
