# Supplementary material for: Household costs, catastrophic out-of-pocket payments and impoverishment related to accessing surgical care in rural Ethiopia
Source: PLoS One. 2026 Feb 6;21(2):e0294215. doi: 10.1371/journal.pone.0294215 (PMC12880665; doi:10.1371/journal.pone.0294215)
Supplement: S3 Table — (DOCX) [file pone.0294215.s003.docx]

**Supplementary table 3: Sources of payment for surgical care**

| Sources of payment | Frequency | Percentage |
| --- | --- | --- |
| Income | 97 | 53.3 |
| Sold items | 69 | 37.9 |
| Relatives | 28 | 15.3 |
| Reimbursement | 13 | 7.1 |
| Saving | 12 | 6.5 |
| Borrowing | 9 | 4.9 |
| Others | 16 | 8.8 |

Total % is more than 100 as payment is made by more than one source
