# Supplementary material for: Household costs, catastrophic out-of-pocket payments and impoverishment related to accessing surgical care in rural Ethiopia
Source: PLoS One. 2026 Feb 6;21(2):e0294215. doi: 10.1371/journal.pone.0294215 (PMC12880665; doi:10.1371/journal.pone.0294215)
Supplement: S4 Table — (DOCX) [file pone.0294215.s004.docx]

**Supplementary File 4: Equations for measuring catastrophic out-of-pocket health payments**

CH = $\frac{1}{N}\sum$ CI (1)

Where CH is the catastrophic headcount (incidence) and CI is the catastrophic index. In this study if a household’s OOP health care expenditure as a proportion of ≥10% or 25% of total consumption, then CIi = 1; otherwise CIi = 0: N is the total number of households in the sample.

The CH estimates the proportion of households that have OOP health care expenditure above the threshold. However, this index does not measure the amount (intensity) by which these payments exceed the chosen threshold.The catastrophic payment overshoot is estimated to give an indication of how much OOP health care expenditure exceed the threshold. The overshoot (O) is estimated as follows:

Oi = CIi ($\frac{\mathrm{Ti}}{\mathrm{Xi}}$ - Z) (2)

Where T_i_ is the OOP payments of household i, X_i_ is the household consumption expenditure (food or nonfood) and Z is the threshold budget share. Following this estimation, the average overshoot is:

O= $\frac{1}{N}\sum$Oi (3)

Therefore, the intensity of catastrophic OOP health care expenditure is computed by averaging the catastrophic overshoot over all households that exceed the catastrophic threshold. This measure is referred to as the mean positive overshoot (MPO), and is computed as follows:

MPO = $\frac{O}{\mathrm{CH}}$ (4)

**Measures of out-of-pocket health care expenditure and impoverishment**

A framework for examining the impact of out-of-pocket health care expenditure was based on the two basic measures of poverty; the headcount and the poverty gap. Accordingly, we measured whether the effect of OOP health care expenditures could lead to poverty using three measures, as recommended by Wagstaff and van Doorslaer: (i) poverty head count, which is the proportion of households living below the poverty line, (ii) poverty gap, the amount by which the poor households fall short of reaching the poverty line and (iii) normalized poverty gap, obtained by dividing the poverty gap by the poverty line.

Estimating these three measures requires setting a poverty line and assessing the extent to which OOP health care expenditure push households below it. The poverty line was set based on median consumption expenditure, a relative poverty line of half and two-thirds of median consumption per capita [35, 36] This figure was used to estimate the poverty headcount, the gap and the normalized gap before (pre) and after (post) health care payments.

The following step was undertaken for measuring impoverishment as a result of OOP health expenditure in the study populations.

Let *zpre*be the pre-payment poverty line and x_i_ be household i's pre-payment consumption expenditure. Then a poor household is indicated by *ppre*=1 if x_<_*zpre*, and zero otherwise.The *pre-payment poverty headcount ratio* is simply the sum of all poor individuals expressed as a fraction of the total population, and defined as:

$H^{pre}=\frac{1}{N}\sum_{i=1}^{N} P_{i}^{pre}$ (1.1)

Where *N* is the sample size. If we denote the individual pre-payment poverty gap by

g_i_*pre*=*p*_i_ *pre*(*zpre_-_* $Xi$), then the average *pre-payment poverty gap* is defined as:

$G^{pre}$=$\frac{1}{N}\sum_{i=1}^{N} {gi}^{pre}$ (1.2)

A *normalized pre-payment poverty gap* expresses the total gap as multiples of the poverty line chosen and is defined as:

*NGpre* =$\frac{G^{pre}}{Z^{pre}}$ (1.3)

The *mean positive pre-payment poverty gap* defines the mean consumption shortfall of the poor households as:

*MPGpre* =$\frac{G^{pre}}{H^{pre}}$ (1.4)

In other words: the average (pre-payment) poverty gap equals the headcount times the mean positive gap. Replacing the pre-payment poverty line *zpre*by the post-payment poverty line *zpost*, and all other superscripts ‘pre’ by the superscript ‘post’ gives the analogous post-payment measures. Therefore:

The *post-payment poverty headcount ratio* is defined as:

$H^{post}=\frac{1}{N}\sum_{i=1}^{N} P_{i}^{post}$ (2.1)

The average *post-payment poverty gap* is

$G^{post}$=$\frac{1}{N}\sum_{i=1}^{N} {gi}^{post}$ (2.2)

A *normalized post -payment poverty gap* is defined as

*NGpost*=$\frac{G^{post}}{Z^{post}}$ (2.3)

The *mean positive post-payment poverty gap*

*MPGpost* =$\frac{G^{post}}{H^{post}}$ (2.4)

Overall, measures of poverty impact (PI)of OOP health payments are then simply defined as the difference between the relevant pre-payment and post-payment measures for the headcount, the poverty gap and the normalized gap.
