# Supplementary material for: Household costs, catastrophic out-of-pocket payments and impoverishment related to accessing surgical care in rural Ethiopia
Source: PLoS One. 2026 Feb 6;21(2):e0294215. doi: 10.1371/journal.pone.0294215 (PMC12880665; doi:10.1371/journal.pone.0294215)
Supplement: S5 Table — (DOCX) [file pone.0294215.s005.docx]

**Supplementary table 5: Types and percentage of surgical operation performed (N=182)**

| Type of surgery | Frequency | Percent |
| --- | --- | --- |
| Caesarean Section | 115 | 63.2 |
| Obstetric emergency | 5 | 2.8 |
| Non-emergency gynaecological | 20 | 11.0 |
| Appendicectomy | 9 | 5.0 |
| Thyroidectomy | 6 | 3.3 |
| Laparotomy | 8 | 4.4 |
| Tonsillectomy | 3 | 1.7 |
| Hernia | 5 | 2.8 |
| Cholecystectomy | 2 | 1.1 |
| Urological | 8 | 4.4 |
| Elective sigmoidectomy | 1 | 0.6 |
| Total | 182 | 100% |
