# Supplementary material for: Household costs, catastrophic out-of-pocket payments and impoverishment related to accessing surgical care in rural Ethiopia
Source: PLoS One. 2026 Feb 6;21(2):e0294215. doi: 10.1371/journal.pone.0294215 (PMC12880665; doi:10.1371/journal.pone.0294215)
Supplement: Attachment — Submitted filename: Response to reviewers_2025_09_01.docx [file pone.0294215.s009.docx]

1^st^ September 2025

Dr Emily Chenette

Editor-in-chief, PLoS One Journal

Dear Dr Chenette

Re:	Revision of submitted manuscript

**PONE-D-23-35112R1**

**Household costs, catastrophic out-of-pocket payments and impoverishment related to accessing surgical care in rural Ethiopia**

We are grateful for the further comments from reviewers. Please see below for full responses to each reviewer point. Please let us know if you require any further clarifications.

We look forward to hearing from you.

Yours sincerely

Professor Charlotte Hanlon

|  | REVIEWER COMMENT | OUR RESPONSE |
| --- | --- | --- |
| # | Reviewer #1 |  |
| 1 | The authors have mentioned that “Households were forced to use hardship coping mechanisms”, I was looking for if there is anything discussed in the manuscript on what is hardship coping mechanisms but did not find anything. This should be clarified | Thank you very much for the valuable and constructive comments. Borrowing and selling assets are considered as hardship coping mechanisms. We clarified this in the following statement:  “Moreover, the other coping strategies used were borrowing and selling assets *(i.e., hardship coping mechanisms)*, which are deeply regressive and could induce long-term household economic impacts and impoverishment” (pp27, lines 474-475) |
| 2 | I would suggest rather than mentioning just “Probably the frequent stock out of medicines and medical supplies may force patients to buy these items from private retail pharmacies”, the authors can check which OOP expenditure item was the highest for obstetric care and support the findings by reference previous literature. | Table 4 provides the direct medical cost. For obstetric care, medicine, supplies and services accounted for 38.8% of this cost supporting our argument. We explained now this as ”Probably the frequent stock out of medicines and medical supplies in government facilities may force patients to buy these items from private retail pharmacies. This has been also reported in other studies [40.41]”  We have now included these references. PP 24 lines 395-396. |
| 3 | The authors have clearly responded to the comment 10, however, the same explanation need to be included in the manuscript as potential reasons for OOP payments despite the care are free. I did not see similar explanation. | Responded above under No 2. |
| 4 | The author could also mention at somewhere how the obstetric care is free, is it the government policy or under insurance/protection scheme? If it is any insurance scheme who pays the premium? | It is a government policy and this was stated on PP 5 lines 92-98  “In Ethiopia pre-payment and other financial risk protection mechanisms are limited. In recognition of this, in recent years the country has introduced health care financing reforms to increase affordable access to health services and achieve universal health coverage (UHC) [8]. Efforts to enhance financial risk protection for people accessing essential health services in Ethiopia include provision of high-impact interventions free of charge through an exemption program”.  Moreover, this was also mentioned on PP24 lines 377-379  In Ethiopia, surgical services are based on payments at the point of care, with the exception of obstetric services which are meant to be provided free of charge through the exemption programme.’ |
| 5 | The author could provide a supplementary table on the type of surgical patients they interviewed beside categorizing them by obstetric and non-obstetric patients. This will be useful to get information on the costs of different types of surgery and understand. | We have added a supplementary table as suggested and added the following text on PP13 lines 253-255.  “The frequencies of differing types of surgical operation are presented in Supplementary File 5: 66% were obstetric surgical procedures.” |
| 6 | A few major costs items can be also provided in US$ for international comparison. | This was addressed in the foot note of the tables that provide costs. Showing *1 USD = 29.07 Birr (2019, purchasing power parity, 1 international dollars=10.74 Birr)* |
|  | Reviewer #2: |  |
| 1 | Thank you for the opportunity to review this manuscript. Comments raised by previous reviewers have been addressed well by the authors. They have clarified certain queries as well as acknowledged limitations to the study that were pointed out. The study objectives, methods, results, and conclusions are well aligned with each other. The methodology used is appropriate based on feasibility. All the financial outcomes, both direct and indirect, have been rigorously documented and analysed. | Thank you very much |
